# Associations of serum folate and vitamin B_12_ levels with all-cause mortality among patients with metabolic dysfunction associated steatotic liver disease: a prospective cohort study

**DOI:** 10.3389/fendo.2024.1426103

**Published:** 2024-12-05

**Authors:** Jiaxin Zhu, Xinyi Liao, Lei Du, Pengju Lv, Jian Deng

**Affiliations:** ^1^ Department of Clinical Laboratory, Zhengzhou Central Hospital Affiliated to Zhengzhou University, Zhengzhou, China; ^2^ Department of Anesthesiology, West China Hospital, Sichuan University, Chengdu, China; ^3^ Department of Thyroid Breast Surgery, The Second Affiliated Hospital, Hengyang Medical School, University of South China, Hengyang, China

**Keywords:** metabolic dysfunction-associated steatotic liver disease, folate, vitamin B12, all-cause mortality, cohort study

## Abstract

**Introduction:**

Serum folate and vitamin B_12_ levels correlate with the prevalence of fatty liver disease, but it is not clear how they affect mortality. Therefore, this study aimed to investigate the association of serum folate and vitamin B_12_ concentrations with all-cause mortality in individuals with metabolic dysfunction-associated steatotic liver disease (MASLD).

**Methods:**

MASLD subjects were from the Third National Health and Nutrition Examination Survey (NHANES III) in the United States, and mortality follow-up data were obtained by linkage to death records from the National Death Index. Multivariable Cox proportional regression models and restricted cubic spline (RCS) models were used to evaluate the association of serum folate/vitamin B_12_ with all-cause mortality in the MASLD population.

**Results:**

3,636 and 2,125 MASLD individuals were included in the analyses related to serum folate and vitamin B_12_, respectively. During a follow-up period of more than 20 years, the RCS models demonstrated significant nonlinear associations of both serum folate (*P <*0.001) and vitamin B_12_ (*P* =0.016) with all-cause mortality in MASLD. When their serum concentrations were below the median level, the risk of all-cause mortality decreased with increasing concentration, reaching a lowest risk around the median level, and then leveled off. In the multivariable cox regression model, for vitamin B_12_, the risk of all-cause mortality was reduced by 42% and 28% in the third and fourth quartile groups, respectively, compared with the lowest quartile group (hazard ratio [HR]=0.58, 95% CI: 0.39-0.86, *P* =0.008; HR =0.72, 95% CI: 0.54-0.96, *P*=0.026, respectively). For folate, the risk of all-cause mortality was reduced by 28% in the third quartile compared with the lowest quartile (HR =0.72, 95% CI: 0.57-0.91, *P* =0.005).

**Conclusion:**

This longitudinal cohort study suggests that low serum folate and vitamin B_12_ levels in patients with MASLD are significantly associated with an elevated risk of all-cause mortality.

## Introduction

Metabolic dysfunction-associated steatotic liver disease (MASLD) is a syndrome of liver disease associated with cardiometabolic dysregulation, previously known as non-alcoholic fatty liver disease (NAFLD) (1). Because of its potential stigmatization and ambiguity in determining etiology, the term NAFLD has been widely criticized (1, 2). Therefore, a new nomenclature framework for fatty liver disease has recently been proposed, in which MASLD is introduced as a new term to replace NAFLD and accompanied by modified diagnostic criteria (1). As the predominant type of steatotic liver disease, the worldwide prevalence of MASLD has been estimated to be more than 30% (3). Alarmingly, MASLD is the leading cause of hepatocellular carcinoma and cirrhosis and is associated with a significant increase in all-cause mortality (4–6). With the rising prevalence of obesity, the incidence of MASLD continues to increase and has resulted in a huge global disease burden (7). The mechanisms underlying the pathogenesis and progression of MASLD are not fully understood; as a multifactorial disease, it is closely related to insulin resistance, oxidative stress, lipid metabolism, and lifestyle environmental factors (8). Although hundreds of drugs are being developed for fatty liver disease, only resmetirom has recently been approved for the treatment of non-alcoholic steatohepatitis, a subtype of NAFLD characterized by hepatitis and liver damage (9). Lifestyle interventions based on exercise and diet modification remain the cornerstone of MASLD management (8, 10). Therefore, it is particularly important to explore new targets for intervention and to identify biomarkers that can be used for risk management in this chronic disease.

Folate, as an important B vitamin, mediates one carbon (1C) metabolic reactions that play a key role in a variety of physiological processes in the body, including nucleic acid and protein synthesis, amino acid homeostasis, redox defense, methylation modification, and immune response (11, 12). Folate deficiency has been found to be strongly associated with a variety of systemic conditions such as cancer, cardiovascular disease, and psychiatric disorders (13–16). In addition, previous studies have demonstrated that folate affects oxidative stress, chronic inflammation, and lipid metabolism in the liver, all of which are pathogenic mechanisms of fatty liver (17–19). The liver is the primary processing and storage site for folate and is critical in the maintenance of folate homeostasis throughout the body (20, 21). Similar to folate, vitamin B12 (also known as cobalamin) is an essential water-soluble vitamin for the maintenance of 1C metabolism, and plays an important role in human health and disease (11, 12). As an integral component of 1C metabolism, vitamin B12 has the ability to support the translocation and storage of folate within the cell (11, 12). Deficiencies of both are often known as the cause of megaloblastic anemia (22). In addition, vitamin B12 is a cofactor for methyl malonyl coenzyme A mutase, which regulates the transfer of long-chain fatty acyl-CoA to the mitochondria and influences lipid metabolic pathways (23). The liver is the major storage organ for vitamin B12, and previous studies have shown that vitamin B12 is associated with hepatocellular carcinoma, cirrhosis, and hepatitis and can independently predict the histologic severity of non-alcoholic steatohepatitis (24–26).

In fact, the relationship between serum folate/vitamin B12 and NAFLD has been explored in several cross-sectional studies. Previous studies have found that serum folate and vitamin B12 are inversely associated with the prevalence of NAFLD and that their levels are lower in patients with NAFLD than in healthy controls (27–30). These studies suggest that folate and vitamin B12 may be involved in the onset and progression of NAFLD. However, no studies have examined the effect of these two essential B vitamins on mortality in patients with NAFLD. Furthermore, for MASLD, this recently proposed concept, its association with serum folate and vitamin B12 has not been reported. To address this research gap, we therefore analyzed the association of serum folate and vitamin B12 levels with long-term all-cause mortality among patients with MASLD in a nationally representative prospective cohort of U.S. adults.

## Method

### Study population

The third National Health and Nutrition Examination Survey (NHANES III) was a major project conducted by the U.S. National Center for Health Statistics from 1988 to 1994 aimed at providing national estimates of the nutritional and health status of children and adults in the United States (31, 32). To enable the survey population to be nationally representative, NHANES III utilized a stratified multistage complex sampling design (31, 32). NHANES III has been frequently used as an unbiased and high-quality dataset for studies in the field of fatty liver disease (33–38). There are other cycles of NHANES that were not included because hepatic imaging data were not available or the linked follow-up period was insufficient. The study population included in the current study was participants aged 20-74 years who underwent hepatic ultrasound. NHANES III has been approved by the NCHS Institutional Review Board. Documented consent was obtained from all participants (https://www.cdc.gov/nchs/nhanes/irba98.htm). The data that we used were completely de-identified for participants, thus exempting the institutional review board.

### Definition of MASLD

The procedure for detecting hepatic steatosis based on hepatic/gallbladder ultrasound is described in detail in the Hepatic Steatosis Ultrasound Images Assessment Procedures Manual of NHANES III (39, 40). Briefly, gallbladder ultrasound was performed on all adults aged 20-74 years who received examinations at the mobile examination center. Hepatic steatosis was assessed based on the following five criteria: (a) liver to kidney contrast; (b) parenchymal brightness; (c) vessel walls definition; (d) deep beam attenuation; and (e) gallbladder wall definition. The original ultrasound video images were reviewed by three ultrasound readers who received standardized training from a board-certified radiologist specializing in liver imaging. Rigorous quality control and quality assurance procedures were used to standardize reading manner among the readers. In the assessment of hepatic steatosis, percentage agreement for intra-rater reliability and inter-rater reliability reached 91.3% and 88.7%, respectively, with kappa coefficients both >0.6 (39). In our study, any degree (mild-severe) of steatosis detected was defined as steatotic liver disease (41–43).

According to the MASLD diagnostic criteria, individuals with steatotic liver disease who had any one of the following five cardiometabolic risk factors were identified as MASLD: (a) body mass index (BMI) ≥25 kg/m^2^, or waist circumference ≥94 cm (male) or ≥80 cm (female); (b) fasting glucose ≥100 mg/dL, or 2-hour post load glucose levels ≥ 140 mg/dl, or hemoglobin A1c ≥ 5.7%, or type 2 diabetes mellitus, or receiving treatment for type 2 diabetes mellitus; (c) blood pressure ≥ 130/85 mmHg or receiving antihypertensive medication; (d) plasma triglycerides ≥ 150 mg/dL or taking lipid-lowering medications; and (e) plasma HDL-cholesterol ≤40 mg/dl (male) or ≤50 mg/dl (female), or taking lipid-lowering medications (1). Steatosis due to underlying etiologies other than cardiometabolic criteria were excluded, including excessive alcohol consumption (alcohol intake ≥30 g/day for males and ≥20 g/day for females), HBV/HCV infection (serum hepatitis B surface antigen-positive or hepatitis C antibody-positive), and iron overload (transferrin saturation ≥50%). Participants who could not be diagnosed with MASLD because of missing data related to the above cardiometabolic risk factors were excluded.

### Measurement of serum folate and victim B12

Both serum folate and vitamin B12 measurements were done by the National Center for Environmental Health using the Bio-Rad Laboratories “Quantaphase II Folate or Folate/B12” radioassay kit (44). The assay was conducted by combining serum samples with ^57^Co- vitamin B12 and ^125^I- folate in a solution that contained dithiothreitol and cyanide. All field-collected specimens were frozen and then transported on dry ice and stored at ≤ -20°C after receipt until analysis. Standard procedures for sample collection, storage, processing, analysis, and quality control are described in detail in Laboratory Procedures Used for the NHANES III (44). The coefficients of variation for the long-term accuracy of the NHANES III assays for serum folate and vitamin B12 were 3-6% (at 3-15 ng/mL) and 5-7% (at 300-1500 pg/mL), respectively (44). Folate or vitamin B12 concentrations below 1% or greater than 99% of the overall distribution were considered outliers and these participants were excluded.

### Clinical and laboratory data

The following socio-demographic data were included: age, sex, race/ethnicity (non-Hispanic white, non-Hispanic black, Mexican-American, other), educational level (≤high school, >high school degree), marital status (married, unmarried), family income to poverty ratio (<1, 1-5, >5), smoking status (current smoker, ex-smoker, never smoker), physical activity (active, median, inactive), Healthy Eating Index, and self-reported general health (excellent, very good, good, fair, poor). These data were derived from the baseline questionnaire interviews. For physical activity, leisure-time activities (such as jogging, swimming, riding, calisthenics, and dancing) were categorized into moderate (MET 3-6) and vigorous (MET > 6) types based on intensity ratings (45). The active physical activity level group was defined as engaging in moderate activities at least five times or vigorous activities at least three times per week; the inactive group was defined as no leisure time physical activity; and the moderate group was participants whose physical activity level fell between the active and inactive groups (46). The Healthy Eating Index is an indicator developed by the U.S. Department of Agriculture to measure the overall quality of an individual’s diet, with scores ranging from 0 to 100 (47). In addition to folate and vitamin B12, laboratory tests included as covariates included FIB-4 index, serum triglycerides, and C-reactive protein. FIB-4 was calculated as “(age (years) × AST (U/L))/((PLT [10^9^/L]) × (ALT (U/L))^1/2^)”. Body measurements body mass index (BMI) and waist circumference were included. Common chronic diseases hypertension, diabetes, and history of heart attack were included. Hypertension was defined as systolic blood pressure ≥ 140 mmHg or diastolic blood pressure ≥ 90 mmHg, or taking antihypertensive medication, or having ever been told a diagnosis of hypertension by a physician. Diabetes mellitus was defined as fasting plasma glucose concentration ≥126 mg/dL, or random/casual plasma glucose concentration ≥200 mg/dL, or Oral Glucose Tolerance Test ≥200 mg/dL, or HbA1c ≥6.5%, or taking antidiabetic medication, or ever been informed of a diagnosis of diabetes by a physician.

### All-cause mortality

Mortality follow-up data were obtained by linking the unique identifiers of participants in NHANES III with death records from the National Death Index. The follow-up period was from the date that the NHANES interview was performed to the occurrence of a death or December 30, 2019, which is the latest data currently available.

### Statistical analysis

Folate and vitamin B12 levels were categorized into four intervals based on quartiles to compare baseline characteristics and mortality status of participants in their respective groups. In comparisons of baseline characteristics, the Rao-Scott chi-squared test was used for dichotomous variables, and the Wilcoxon rank-sum test was used for continuous variables. Cox proportional regression models were applied to examine differences in all-cause mortality among MASLD patients in different serum folate and vitamin B12 quartile groups, with participants in the lowest serum folate and vitamin B12 quartile intervals being used as the reference group, respectively. Since the serum folate and vitamin B12 levels of most MASLD participants fell within the normal ranges suggested by some clinical guidelines for healthy adults (serum folate: 3–20 ng/mL; vitamin B12: 160–950 pg/mL) (48), we did not use the normal ranges for both metrics as a reference. In our analysis, the vast majority of participants in the lowest quartile for serum folate and vitamin B12 were still within the normal range for both markers, with fewer than 10% classified as deficient. Given the differences in sources of folate and vitamin B12, we conducted an analysis based on the combined status of both. Specifically, we classified the levels of each indicator into high-level and low-level groups based on their median values, resulting in the following four combinations: low folate & low vitamin B12 group, low folate & high vitamin B12 group, high folate & low vitamin B12 group, and high folate & high vitamin B12 group. The low folate & low vitamin B12 group was used as the reference group. Age, sex, and race-adjusted models considered age, sex, and race as confounders. Moreover, we developed multivariable Cox models further adjusting for demographic characteristics (educational level, marital status, family income level), lifestyle factors (smoking status, physical activity, Healthy Eating Index, vitamin C intake, and vitamin D intake), body measurements (body mass index, waist circumference), laboratory tests (FIB-4 index, serum triglycerides, C-reactive protein), and health status (self-reported general health, diabetes mellitus, hypertension, history of heart attack). To examine whether the effects of folate and vitamin B12 on mortality in MASLD were age-, sex-, or race-specific, we conducted stratified analyses according to them and analyzed interaction effects. To investigate the dose-response effects of vitamin B12 and folate levels on mortality in patients with MASLD, we used restricted cubic spline (RCS) models adjusted for baseline age, sex, and race/ethnicity, educational level, marital status, family income level, smoking status, physical activity, Healthy Eating Index, FIB-4 index, triglycerides, C-reactive protein, body mass index, waist circumference, self-reported general health, diabetes mellitus, hypertension, and history of heart attack. The respective median values of folate and vitamin B12 were used as reference points.

Sensitivity analyses were performed by excluding participants who died within two years of follow-up to rule out a reverse causal association between folate/vitamin B12 levels and all-cause mortality in patients with MASLD. Primary analyses were repeated (age-, sex-, and race-adjusted Cox regression models, multivariable Cox regression models, and RCS dose-response effect analyses) to examine whether the findings were robust.

We considered the complex survey design of NHANES III and used appropriate sample weights in all statistical analyses to make the results nationally representative. All tests were two-sided and *P*<0.05 was considered statistically significant. R version 4.3.1 (https://www.r-project.org/) was used to perform all statistical analyses.

## Results

**Figure 1:**
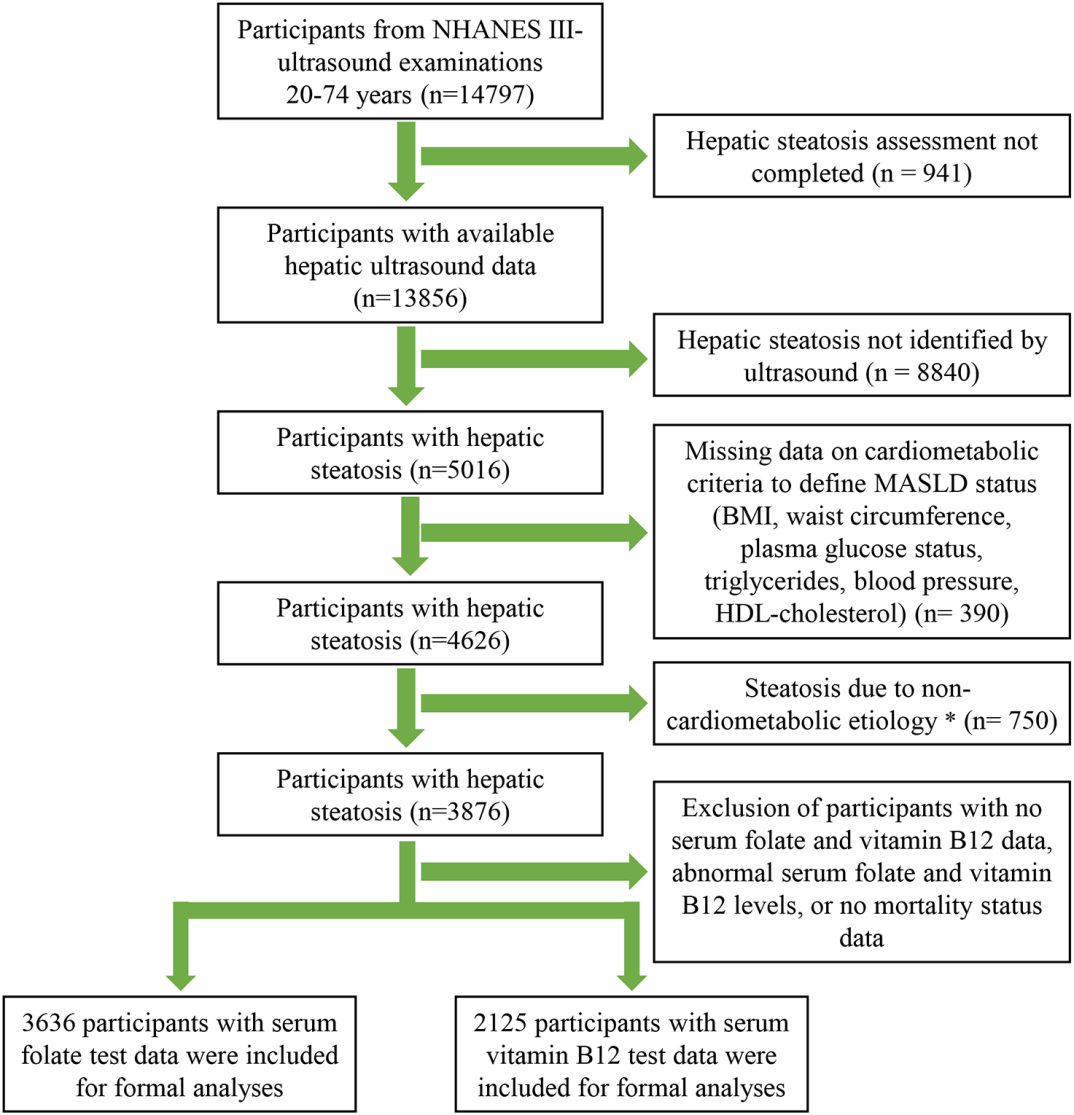
Flow Diagram of Participants Inclusion in the Study. *Alcohol consumption >30 g/day for male and >20 g/day for female, HBV/HCV infection, or serum transferrin saturation >50%.

A total of 14,797 participants underwent an ultrasound examination, of which 941 were excluded because of missing or un-gradable image data. Of the 13,856 participants with available hepatic/gallbladder ultrasound data, hepatic steatosis was detected in 5016 individuals. After excluding participants with non-cardiometabolic etiologies and missing data on cardiometabolism, folate/vitamin B12 levels, and mortality status, 3636 participants with serum folate data and 2125 participants with serum vitamin B12 data were ultimately included in the formal analysis. The detailed study screening process is displayed in **Figure 1**.

For serum folate levels, the concentration ranges of the four groups according to the quartile method were: quartile 1 (<3.3 ng/mL), quartile 2 (3.3-4.7 ng/mL), quartile 3 (4.8-7.3 ng/mL), and quartile 4 (>7.3 ng/mL); for serum vitamin B12 levels, the concentration ranges of the four groups were: quartile 1 (<352 pg/mL), quartile 2 (352-457 pg/mL), quartile 3 (458-588 pg/mL), and quartile 4 (>588 pg/mL). In the serum folate analysis, the mean age of participants was 46.94 (SE: 0.43), and in the serum vitamin B12 analysis, the mean age of participants was 46.34 (SE: 0.71). Interestingly, the age tended to be older in the higher quartile serum folate group (P<0.001), whereas there was no significant difference in age among the different vitamin B12 groups (P=0.943). There was a difference in the gender ratio among the different serum vitamin B12 groups, with the high quartile groups (quartile 3 and quartile 4) tending to have a greater proportion of females (P=0.033), whereas the gender ratio was relatively balanced among the different folate groups (P=0.382). When complex sampling was considered, non-Hispanic whites were the most numerous, and there were differences in the ethnic distributions across the different quartile groups for both serum folate and vitamin B12. Detailed study characteristics of the included population are displayed in **Table 1**.

**Table 1:** Baseline characteristics of participants with MASLD according to the serum folate and vitamin B_12_ levels.

Characteristic	Serum folate level, ng/mL						Serum vitamin B_12_ level, pg/mL					
	Overall	Quartile 1 (<3.3)	Quartile 2 (3.3-4.7)	Quartile 3 (4.8-7.3)	Quartile 4 (>7.3)	*p-*value^a^	Overall	Quartile 1 (<352)	Quartile 2 (352–457)	Quartile 3 (458-588)	Quartile 4 (>588)	*p-*value^a^
**Participants, no**	3636	917	909	926	884		2125	533	532	530	530	
**Age (years)**	46.94 (0.43)	41.28 (0.69)	45.23 (0.68)	47.57 (0.85)	52.23 (0.78)	<0.001	46.34 (0.71)	46.31 (1.32)	46.65 (0.82)	46.13 (1.11)	46.25 (1.11)	0.943
**Gender**						0.382						0.033
Male	1,659 (50.9%)	435 (53.7%)	408 (48.8%)	423 (52.7%)	393 (48.4%)		903 (51.1%)	246 (54.5%)	246 (57.9%)	228 (47.7%)	183 (41.5%)	
Female	1,977 (49.1%)	482 (46.3%)	501 (51.2%)	503 (47.3%)	491 (51.6%)		1,222 (48.9%)	287 (45.5%)	286 (42.1%)	302 (52.3%)	347 (58.5%)	
**Race/ethnicity**						<0.001						<0.001
Non-Hispanic white	1,323 (76.0%)	269 (71.4%)	254 (71.1%)	331 (74.0%)	469 (84.7%)		724 (74.7%)	238 (81.5%)	194 (79.0%)	166 (73.0%)	126 (61.1%)	
Non-Hispanic black	837 (9.0%)	273 (12.8%)	224 (11.8%)	204 (8.4%)	136 (4.6%)		520 (9.0%)	83 (4.3%)	109 (7.4%)	126 (9.0%)	202 (18.0%)	
Mexican-American	1,341 (6.9%)	345 (7.4%)	401 (9.4%)	353 (7.4%)	242 (4.1%)		785 (7.0%)	187 (6.1%)	207 (7.5%)	216 (7.6%)	175 (6.9%)	
Other	135 (8.1%)	30.0 (8.4%)	30 (7.6%)	38 (10.2%)	37 (6.5%)		96 (9.3%)	25 (8.1%)	22 (6.1%)	22 (10.4%)	27 (14.0%)	
**Marital status**						0.499						0.417
Married	2,390 (69.1%)	575 (69.2%)	600 (70.4%)	621 (70.9%)	594 (66.6%)		1,352 (67.8%)	356 (67.7%)	339 (69.5%)	351 (70.3%)	306 (62.6%)	
Unmarried	1,238 (30.9%)	341 (30.8%)	307 (29.6%)	302 (29.1%)	288 (33.4%)		767 (32.2%)	176 (32.3%)	191 (30.5%)	178 (29.7%)	222 (37.4%)	
**PIR**						0.022						0.286
<1	827 (12.2%)	253 (16%)	228 (13.2%)	207 (12.8%)	139 (7.9%)		522 (11.7%)	110 (10.0%)	130 (11.0%)	141 (11.4%)	141 (15.4%)	
1-5	2,204 (72%)	524 (72.1%)	541 (73.0%)	571 (72.6%)	568 (70.7%)		1,261 (70.9%)	340 (71.8%)	308 (69.0%)	306 (69.9%)	307 (73.3%)	
>5	293 (15.8%)	46 (11.8%)	65 (13.9%)	72 (14.6%)	110 (21.4%)		182 (17.4%)	49 (18.2%)	53 (20.1%)	43 (18.6%)	37 (11.2%)	
**Education**						<0.001						0.252
≤High school degree	2,933 (71.0%)	785 (78.5%)	737 (74.1%)	757 (71.0%)	654 (62.5%)		1,705 (69.6%)	423 (65.6%)	424 (69.3%)	429 (72.3%)	429 (72.7%)	
>High school degree	689 (29.0%)	128 (21.5%)	169 (25.9%)	165 (29.0%)	227 (37.5%)		410 (30.4%)	108 (34.4%)	105 (30.7%)	98 (27.7%)	99 (27.3%)	
**Physical activity**						<0.001						0.889
Active	1,175 (38.1%)	231 (29.1%)	283 (35.1%)	312 (41.2%)	349 (44.8%)		691 (39.6%)	166 (39.4%)	176 (38.5%)	176 (42.9%)	173 (37.2%)	
Median	1,555 (44.9%)	411 (50.1%)	410 (49.7%)	393 (41.3%)	341 (40.5%)		889 (43.8%)	226 (43.8%)	223 (45.7%)	218 (41.2%)	222 (44.7%)	
Inactive	906 (17.0%)	275 (20.7%)	216 (15.2%)	221 (17.5%)	194 (14.7%)		545 (16.6%)	141 (16.9%)	133 (15.8%)	136 (15.9%)	135 (18.0%)	
**HEI**	63.93 (0.42)	58.86 (0.61)	60.50 (0.57)	64.54 (0.70)	69.85 (0.67)	<0.001	64.19 (0.67)	63.74 (0.79)	64.26 (0.97)	62.94 (1.37)	66.31 (0.96)	0.179
**Smoking status**						<0.001						0.029
Current smoker	738 (21.9%)	301 (36.2%)	180 (21.6%)	164 (20.7%)	93 (11.7%)		414 (21.2%)	101 (21.1%)	119 (25.3%)	102 (22.4%)	92 (14.6%)	
Ex-smoker	1,056 (32.3%)	197 (25.5%)	260 (32.2%)	275 (30.8%)	324 (39.2%)		573 (31.9%)	142 (26.5%)	153 (36.8%)	135 (33.0%)	143 (32.4%)	
Never smoker	1,841 (45.8%)	419 (38.3%)	468 (46.2%)	487 (48.5%)	467 (49.2%)		1,138 (46.9%)	290 (52.4%)	260 (37.9%)	293 (44.6%)	295 (53.0%)	
**BMI**	30.01 (0.26)	30.90 (0.47)	30.16 (0.31)	30.11 (0.43)	29.08 (0.39)	0.003	30.03 (0.35)	29.61 (0.47)	29.82 (0.36)	30.72 (0.65)	30.08 (0.68)	0.532
**Waist circumference**	100.96 (0.61)	102.35 (0.96)	101.37 (0.75)	101.70 (1.08)	98.90 (0.76)	0.004	100.53 (0.80)	101.07 (1.12)	101.52 (0.89)	100.47 (1.67)	98.51 (1.12)	0.104
**Diabetes**	975 (20.1%)	185 (13.6%)	225 (18.6%)	269 (21.0%)	296 (25.6%)	<0.001	563 (19.4%)	123 (17.8%)	134 (18.0%)	133 (19.2%)	173 (23.6%)	0.487
**Hypertension**	1,587 (42.3%)	364 (38.9%)	367 (39.1%)	433 (45.9%)	423 (44.0%)	0.116	895 (39.8%)	235 (46.2%)	199 (32.6%)	222 (38.1%)	239 (41.9%)	0.050
**Heart attack**	185 (4.5%)	38 (3.4%)	38 (3.3%)	47 (4.2%)	62 (6.6%)	0.055	90 (3.8%)	24 (3.5%)	19 (4.4%)	21 (3.3%)	26 (3.8%)	0.828
**Self-reported general health**						0.163						0.642
Excellent	429 (15.6%)	96 (14.2%)	96 (15.1%)	106 (12.7%)	131 (19.6%)		246 (16.0%)	69 (19.4%)	53 (12.6%)	66 (15.6%)	58 (15.8%)	
Very good	774 (29.6%)	200 (30.2%)	177 (28.3%)	195 (31.2%)	202 (28.8%)		468 (31.1%)	128 (30.7%)	122 (34.2%)	110 (30.3%)	108 (28.8%)	
Good	1,389 (36.4%)	356 (38.0%)	364 (34.5%)	356 (38.5%)	313 (34.6%)		815 (36.2%)	195 (33.9%)	223 (38.8%)	203 (37.2%)	194 (34.8%)	
Fair	850 (15.1%)	216 (14.5%)	228 (19.5%)	216 (13.4%)	190 (14.1%)		491 (14.2%)	122 (13.9%)	109 (12.8%)	127 (13.4%)	133 (17.2%)	
Poor	193 (3.2%)	49 (3.0%)	43 (2.6%)	53 (4.2%)	48 (2.9%)		105 (2.6%)	19 (2.1%)	25 (1.6%)	24 (3.5%)	37 (3.4%)	
**FIB-4**	0.95 (0.01)	0.78 (0.02)	0.88 (0.02)	0.96 (0.02)	1.14 (0.03)	<0.001	0.94 (0.02)	0.97 (0.03)	0.92 (0.03)	0.92 (0.04)	0.94 (0.05)	0.815
**TGP**	194.72 (3.89)	192.05 (7.67)	181.68 (6.39)	196.41 (11.55)	204.69 (9.08)	0.167	200.12 (5.54)	199.17 (9.60)	188.32 (10.88)	224.18 (20.28)	186.30 (9.46)	0.730
**CRP**	0.47 (0.02)	0.48 (0.02)	0.46 (0.02)	0.47 (0.04)	0.47 (0.03)	0.237	0.49 (0.02)	0.43 (0.02)	0.50 (0.05)	0.50 (0.04)	0.52 (0.04)	0.414
**Vitamin C intake (mg)**	37.57 (1.68)	26.55 (1.81)	34.28 (3.61)	38.82 (2.71)	47.30 (2.71)	<0.001	35.32 (2.24)	31.47 (2.91)	38.72 (3.02)	37.31 (4.07)	33.94 (3.62)	0.488
**Vitamin E intake (mg)**	5.18 (0.26)	3.75 (0.32)	4.80 (0.42)	4.34 (0.24)	7.24 (0.62)	0.001	5.52 (0.36)	5.39 (0.42)	4.70 (0.42)	6.42 (0.95)	5.66 (0.66)	0.619

a For categorical variables, *p*-value was calculated by Rao-Scott chi-squared test; for continuous variables, Wilcoxon rank-sum test for complex survey samples was used.

MASLD, Metabolic dysfunction associated steatotic liver disease; HEI, Healthy eating index; PIR, Family income to poverty ratio; BMI, body mass index; CRP, C-reactive protein; TGP, serum triglycerides.

FIB-4 was calculated from “(age (years) × AST (U/L))/((PLT [10^9^/L]) × (ALT (U/L))^1/2^)”.

All estimates account for complex survey designs, and data are shown as weighted means (standard errors) or percentages as appropriate.

### Serum folate and mortality

**Figure 2:**
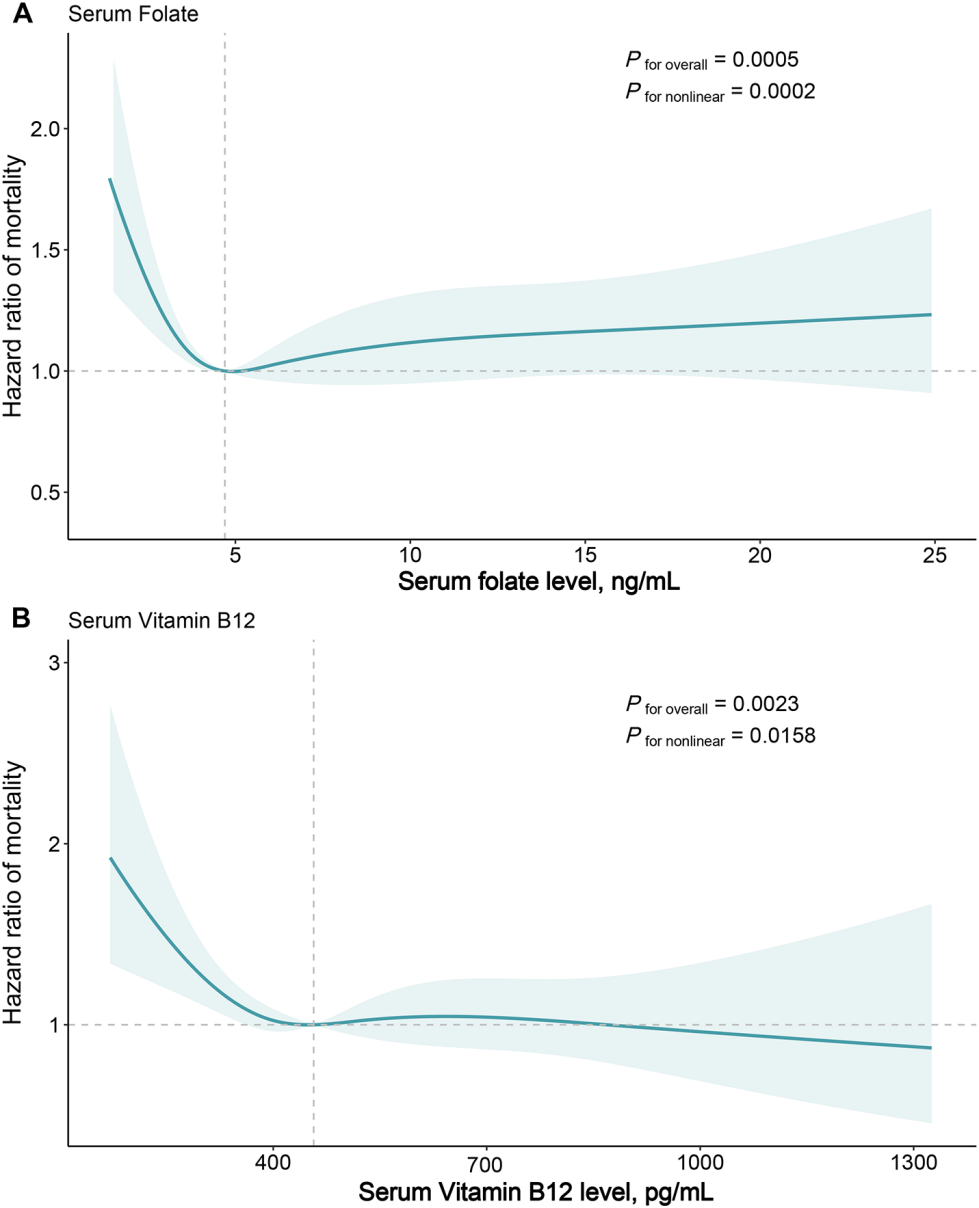
Dose-response Association of Serum Folate **(A)** and Vitamin B_12_ **(B)** Levels with All-cause Mortality in Patients with Metabolic Dysfunction-associated Fatty Liver Disease. Hazard ratios were estimated by multivariable restricted cubic spline models, with knots placed at 5th, 35th, 65th, and 95th percentiles. Solid line represents hazard ratios and shaded areas represents 95% CIs. The reference points are the median values for serum folate (4.7 ng/mL) and serum vitamin B12 (457.0 pg/mL) level. Risk estimates were adjusted for baseline age, sex, and race/ethnicity, educational level, marital status, family income level, smoking status, physical activity, Healthy Eating Index, FIB-4 index, triglycerides, C-reactive protein, body mass index, waist circumference, self-reported general health, diabetes mellitus, hypertension, and history of heart attack.

During a median 26.08 (IQR:18.08-28) years of follow-up for 3636 participants, 1571 deaths occurred. In age, sex, race-adjusted Cox proportional regression models, the higher folate quartile had a significantly lower risk of mortality compared with the lowest quartile group, with HRs of 0.77 (95% CI: 0.60-0.97, *P*=0.026) for quartile 2, 0.68 for quartile 3 (95% CI: 0.56-0.82, *P*<0.001), and 0.74 (95% CI: 0.59-0.94, *P*=0.011) for quartile 4 (**Table 2**). In multivariable models further adjusted for other potential confounders, only the quartile 3 group showed a significant reduction in mortality (HR=0.72, 95% CI: 0.57-0.91, *P*=0.005) (**Table 2**). The multivariable RCS dose-response analysis showed a significant nonlinear association between serum folate levels and all-cause mortality in patients with MASLD (*P*_for nonlinear <_0.001) (**Figure 2A**). At serum folate levels less than the median value, the risk of all-cause mortality decreased with increasing folate levels, reaching a lowest risk at around 4.7 ng/mL and then slowly increasing.

**Table 2:** Hazard ratios of all-cause mortality by serum folate and vitamin B_12_ levels among adults with MASLD.

	Age, sex, race-adjusted model^a^		Multivariate model^b^	
	HR (95% CIs)	*P-value*	HR (95% CIs)	*P-value*
Serum Folate				
Quartile 1	1 (reference)		1 (reference)	
Quartile 2	0.77 (0.60-0.97)	0.026	0.81 (0.61-1.06)	0.128
Quartile 3	0.68 (0.56-0.82)	<0.001	0.72 (0.57-0.91)	0.005
Quartile 4	0.74 (0.59-0.94)	0.011	0.89 (0.69-1.15)	0.369
Serum Vitamin B12				
Quartile 1	1 (reference)		1 (reference)	
Quartile 2	0.83 (0.59-1.17)	0.293	0.73 (0.52- 1.03)	0.074
Quartile 3	0.77 (0.54-1.08)	0.128	0.58 (0.39-0.86)	0.008
Quartile 4	0.90 (0.68-1.20)	0.476	0.72 (0.54-0.96)	0.026
Serum Folate & Vitamin B12*				
Low folate & low vitamin B12	1 (reference)		1 (reference)	
Low folate & high vitamin B12	0.95 (0.65-1.39)	0.795	0.79 (0.51-1.22)	0.295
High folate & low vitamin B12	0.78 (0.56-1.09)	0.152	0.84 (0.60-1.18)	0.321
High folate & high vitamin B12	0.71 (0.52-0.97)	0.031	0.64 (0.45-0.89)	0.009

a Multivariable Cox proportional regression analysis adjusted for age, sex, and race/ethnicity;

b Multivariable Cox proportional regression analysis adjusted for age, sex, race/ethnicity, educational level, marital status, family income level, smoking status, physical activity, Healthy Eating Index, FIB-4 index, serum triglycerides, C-reactive protein, body mass index, waist circumference, self-reported general health, diabetes mellitus, hypertension, history of heart attack, vitamin C intake, and vitamin E intake.

*Serum folate and serum vitamin B12 levels were considered simultaneously. Values below the median were classified as low levels, and values above the median were classified as high levels. Based on these two serum indicators, patients were divided into four groups: low folate & low vitamin B12 group, low folate & high vitamin B12 group, high folate & low vitamin B12 group, and high folate & high vitamin B12 group.

MASLD, Metabolic dysfunction associated steatotic liver disease; HR, hazard ratio; CIs, confidence intervals.

### Serum vitamin B_12_ and mortality

2125 participants incurred 835 deaths during a median 25.75 (IQR:19.25-26.83) years of follow-up. The age, sex, race-adjusted Cox proportional regression models indicated that higher vitamin B12 quartiles tended to have a lower risk of mortality compared with the lowest quartile group, but did not reach statistical significance (**Table 2**). After further adjustment for potential confounders, the multivariable Cox model indicated that the quartile 3 (HR=0.58, 95% CI: 0.39-0.86, *P*=0.008) and quartile 4 (HR=0.72, 95% CI: 0.54-0.96, *P*=0.026) groups had a significantly lower risk of mortality compared with the lowest quartile group (**Table 2**). Similar to folate, multivariable RCS models showed a significant nonlinear correlation between serum vitamin B12 concentrations and all-cause mortality (*P*_for nonlinear_ =0.016), with the risk of mortality decreasing with increasing vitamin B12 levels when serum vitamin B12 concentrations were below the median value, reaching a nadir risk at around 457 pg/mL. In contrast, the change in risk of all-cause mortality was relatively smooth after serum vitamin B12 concentrations were greater than the median value (**Figure 2B**).

### Serum folate and vitamin B_12_ combination status and mortality

Considering the significant differences in the sources of serum folate and vitamin B12, we next evaluated the combined effects of serum folate and vitamin B12 on MASLD population. In multivariable models adjusted for full potential confounders, compared to the low folate & low vitamin B12 group, both the low folate & high vitamin B12 group and the high folate & low vitamin B12 group tended to have lower all-cause mortality (HR=0.79, 95% CI: 0.51-1.22, and HR=0.84, 95% CI: 0.60-1.18, respectively), although these differences were not statistically significant (**Table 2**). Interestingly, the high folate & high vitamin B12 group showed a significantly reduced mortality (HR=0.64, 95% CI: 0.45-0.89, *P*=0.009) (**Table 2**).

### Stratified analysis

In stratified analyses according to age, sex, and race, no significant interaction effects were found for them on the correlation between folate/vitamin b12 and all-cause mortality (**Tables 3**, **4**). However, there were some scenarios of marginal statistical significance. Specifically, the association between folate and mortality in patients with MASLD appeared to be more significant in females (*P*=0.098). Serum folate was statistically correlated with reduced all-cause mortality in the fourth and third quartiles of the young and middle-aged groups, respectively (**Table 3**). In the analysis of vitamin b12, the association between elevated serum vitamin b12 concentrations and reduced mortality appeared to be more significant in middle-aged and older adults (*P* =0.071) (**Table 4**). In the combined analysis of vitamin B12 and folate, we found that Non-Hispanic Black individuals did not seem to benefit from the simultaneous elevation of both (**Table 5**).

**Table 3:** Association of serum folate levels with all-cause mortality in different subgroups of patients with MASLD.

	HR (95% CIs) by quartile^a^				*P-value*_for interaction_
	Quartile 1	Quartile 2	Quartile 3	Quartile 4	
Gender					0.098
Male	1 (reference)	1.16 (0.76-1.78)	0.88 (0.59-1.30)	1.19 (0.81-1.76)	
Female	1 (reference)	0.56 (0.42-0.74)	0.59 (0.46-0.77)	0.74 (0.56-0.98)	
Age					0.124
20-39 years	1 (reference)	0.90 (0.29-2.83)	0.57 (0.28-1.15)	0.17 (0.07-0.43)	
40-59 years	1 (reference)	0.77 (0.48-1.22)	0.60 (0.38-0.95)	1.01 (0.61-1.67)	
60-74 years	1 (reference)	0.97 (0.68-1.37)	0.96 (0.72-1.28)	1.10 (0.83-1.44)	
Race/ethnicity					0.415
Non-Hispanic white	1 (reference)	0.82 (0.57-1.18)	0.72 (0.55-0.94)	0.91 (0.68-1.21)	
Non-Hispanic black	1 (reference)	0.88 (0.56-1.40)	0.87 (0.57-1.32)	1.30 (0.85-1.99)	
Mexican-American	1 (reference)	0.73 (0.50-1.06)	0.90 (0.57-1.43)	0.93 (0.57- 1.58)	
Other	1 (reference)	1.17 (0.23-5.93)	0.14 (0.03-0.64)	0.07 (0.02-0.33)	

a Multivariable Cox proportional regression analysis adjusted for age, sex, and race/ethnicity, educational level, marital status, family income level, smoking status, physical activity, Healthy Eating Index, FIB-4 index, serum triglycerides, C-reactive protein, body mass index, waist circumference, self-reported general health, diabetes mellitus, hypertension, history of heart attack, vitamin C intake, and vitamin E intake.

MASLD, Metabolic dysfunction associated steatotic liver disease; HR, hazard ratio; CIs, confidence intervals.

**Table 4:** Association of serum vitamin B_12_ levels with all-cause mortality in different subgroups of patients with MASLD.

	HR (95% CIs) by quartile^a^				*P-value*_for interaction_
	Quartile 1	Quartile 2	Quartile 3	Quartile 4	
Gender					0.412
Male	1 (reference)	0.88 (0.61-1.29)	0.81 (0.54-1.21)	0.79 (0.52-1.20)	
Female	1 (reference)	0.55 (0.31-0.98)	0.47 (0.26-0.87)	0.61 (0.38-1.00)	
Age					0.071
20-39 years	1 (reference)	1.78 (0.51-6.15)	1.68 (0.62-4.59)	2.47 (0.53-11.5)	
40-59 years	1 (reference)	0.66 (0.34-1.30)	0.56 (0.28-1.12)	0.58 (0.31-1.06)	
60-74 years	1 (reference)	0.67 (0.47-0.96)	0.50 (0.33-0.75)	0.65 (0.47-0.90)	
Race/ethnicity					0.498
Non-Hispanic white	1 (reference)	0.74 (0.50- 1.09)	0.55 (0.36- 0.82)	0.70 (0.51- 0.97)	
Non-Hispanic black	1 (reference)	0.60 (0.33- 1.07)	1.17 (0.65- 2.10)	0.91 (0.53- 1.59)	
Mexican-American	1 (reference)	0.82 (0.59- 1.13)	0.69 (0.42- 1.11)	0.83 (0.50- 1.37)	
Other	1 (reference)	1.35 (0.21- 8.46)	0.25 (0.03- 2.43)	0.10 (0.02- 0.44)	

a Multivariable Cox proportional regression analysis adjusted for age, sex, and race/ethnicity, educational level, marital status, family income level, smoking status, physical activity, Healthy Eating Index, FIB-4 index, serum triglycerides, C-reactive protein, body mass index, waist circumference, self-reported general health, diabetes mellitus, hypertension, history of heart attack, vitamin C intake, and vitamin E intake.

MASLD, Metabolic dysfunction associated steatotic liver disease.

**Table 5:** Association of serum folate & vitamin B_12_ levels with all-cause mortality in different subgroups of patients with MASLD.

	HR (95% CIs) by quartile^a^				*P-value*_for interaction_
	Low folate & low vitamin B12	Low folate & high vitamin B12	High folate & low vitamin B12	High folate & high vitamin B12	
Gender					0.493
Male	1 (reference)	0.78 (0.44-1.37)	0.70 (0.39-1.23)	0.68 (0.42-1.09)	
Female	1 (reference)	0.85 (0.47-1.54)	1.05 (0.73-1.51)	0.64 (0.42-0.98)	
Age					0.679
20-39 years	1 (reference)	1.89 (0.88-4.07)	0.41 (0.14-1.22)	0.36 (0.06-2.00)	
40-59 years	1 (reference)	0.47 (0.22-0.99)	0.55 (0.27-1.12)	0.64 (0.38-1.09)	
60-74 years	1 (reference)	0.66 (0.45-0.98)	0.96 (0.70-1.30)	0.64 (0.47-0.87)	
Race/ethnicity					0.079
Non-Hispanic white	1 (reference)	0.64 (0.38- 1.07)	0.79 (0.53- 1.17)	0.61 (0.41- 0.89)	
Non-Hispanic black	1 (reference)	1.35 (0.89- 2.05)	0.95 (0.53- 1.73)	1.35 (0.79- 2.30)	
Mexican-American	1 (reference)	0.86 (0.51- 1.46)	1.08 (0.52- 2.23)	0.85 (0.43- 1.70)	
Other	1 (reference)	1.28 (0.06- 27.3)	0.31 (0.01- 9.29)	0.06 (0.01- 0.40)	

a Multivariable Cox proportional regression analysis adjusted for age, sex, and race/ethnicity, educational level, marital status, family income level, smoking status, physical activity, Healthy Eating Index, FIB-4 index, serum triglycerides, C-reactive protein, body mass index, waist circumference, self-reported general health, diabetes mellitus, hypertension, history of heart attack, vitamin C intake, and vitamin E intake.

MASLD, Metabolic dysfunction associated steatotic liver disease.

### Sensitivity analysis

We repeated the analyses for the primary findings after excluding participants whose deaths occurred within two years of follow-up. Sensitivity analyses showed that participants who died within a short period of time had little effect on the results of the Cox proportional regression model and the multivariable RCS model, suggesting that the correlation between folate/vitamin b12 and mortality was not confounded by reverse causal effects (**Supplementary Table S1**, **Supplementary Figure S1**).

## Discussion

To our knowledge, this is the first study to explore the effect of serum folate and vitamin B12 levels on all-cause mortality in patients with MASLD. In this large population-based prospective cohort study with a follow-up of more than 20 years, we found that both serum folate and vitamin B12 concentrations were significantly associated with all-cause mortality in individuals with MASLD. Low serum folate and vitamin B12 concentrations implied worse long-term outcomes for individuals with MASLD. Interestingly, we found that participants with both high folate and high vitamin B12 levels exhibited a more pronounced reduction in mortality compared to those with elevated levels of either folate or vitamin B12 alone. Sensitivity analyses confirmed the robustness of these findings from this study.

Of note, the association between serum folate and mortality shows inconsistent trends between men and women with MASLD. Some previous studies have suggested that estrogen influences folate metabolism and effects, but it is unclear whether this interaction contributes to the observed differences in MASLD patients (49, 50). Additionally, the association between serum vitamin B12 and mortality shows opposite trends in individuals under and over 40 years of age. This may be because MASLD patients over 40 tend to have poorer baseline health and more comorbidities, making the antioxidant, anti-inflammatory, and cardiovascular protective roles of vitamin B12 more critical (51, 52). In contrast, in patients under 40, high folate levels do not appear to offer similar protective effects. In summary, current evidence is insufficient to clarify the specific mechanisms of interaction between age/gender and the effects of these two nutrients in MASLD patients. However, it is important to note that the impact of folate and vitamin B12 on patient outcomes is not always consistent across different demographic groups.

Previous studies have shown that patients with NAFLD have lower serum folate and vitamin B12 concentrations compared with the general population (27–30). Furthermore, higher concentrations of folate/vitamin B12 and severity of liver fibrosis, steatosis, and nonalcoholic steatohepatitis were inversely correlated in patients with fatty liver (26, 53, 54). Thus, our findings may be due to the fact that low folate and vitamin B12 concentrations in MASLD imply a more severe disease status. Folate and vitamin B12 may be useful as biomarkers to independently predict mortality in individuals with MASLD, but more evidence from other geographic areas or ethnicities is needed to support this.

Several published studies have investigated the correlation between folate/vitamin B12 concentrations and mortality in the general population, but the results have been inconsistent. For example, Wolffenbuttel et al. showed that low serum vitamin B12 concentrations were significantly associated with increased all-cause mortality (55), but Flores-Guerrero et al. found that high vitamin B12 concentrations represented increased all-cause mortality in the general population of the city of Groningen, the Netherlands (56). In an elderly population in China, the correlation between serum vitamin B12 and all-cause mortality showed a J-shaped pattern (57). Existing studies exploring the association between folate concentrations in the body and mortality in the general population tend to report beneficial health effects of folate (58). For example, Peng et al. and Song et al. found that higher folate levels were associated with lower all-cause and cause-specific mortality (59, 60). Interestingly, several studies have shown that folate intake is associated with a reduced risk of mortality, whereas vitamin B12 intake did not have this effect (55, 61–63).

Some cross-sectional investigations have found an inverse association between folate intake and the prevalence of NAFLD (28, 29). However, there is no evidence from longitudinal studies that increased folate intake reduces the risk of mortality in individuals with fatty liver disease. Although our study suggests that low folate levels *in vivo* may be associated with a higher risk of mortality and studies from the general population have shown the benefits of folate intake, more direct clinical evidence is still needed as to whether folate supplementation can be used as a dietary intervention strategy for patients with MASLD. Compared to vitamin B12 deficiency, folate deficiency is more common because the body stores only a small amount of folate, so when the ingested diet is deficient in folate, the body can exhibit folate deficiency within a few months (64). However, insufficient intake is only part of the reason for folate or vitamin B12 deficiency in the body; low serum concentrations of both nutrients may also represent absorption disorders such as celiac disease, pancreatic disease, small bowel resection, endogenous factor deficiencies, or the effects of certain medications such as metformin, methotrexate, and antibiotics (65). Therefore, further studies are still needed to determine whether folate or vitamin B12 supplementation is truly effective as an intervention.

The potential mechanisms by which folate/vitamin B12 affects mortality in MASLD remain to be elucidated. Both play a core role in 1C metabolism, affecting a wide range of physiological activities such as protein and nucleic acid synthesis, methylation of DNA, and post-translational modification of proteins (11, 12). A recent study found that folate/vitamin B12 reduced circulating concentrations of homocysteine and improved autophagy through transmethylation, while serum homocysteine levels were significantly associated with worse liver inflammation and degree of fibrosis (66). Thus, dietary folate/vitamin B12 supplementation in the mouse model slows the progression of non-alcoholic steatohepatitis and reverses inflammation and fibrosis (66). In addition, folate/vitamin B12 is strongly associated with lipid metabolism (17, 67); low folate/vitamin B12 levels are associated with a high prevalence of metabolic syndromes, and their deficiency increases lipid accumulation in adipocytes, leptin production, and inflammatory factors, and thus may influence the progression of fatty liver (17, 67). Furthermore, deficiencies in vitamin b12 and folate increase oxidative stress by raising homocysteine levels (51, 68–70). Since oxidative stress plays an important role in the progression of fatty liver (71), ensuring adequate levels of vitamin b12 and folate may improve the prognosis of patients through this mechanism.

As the first large-scale investigation into the association of serum folate and vitamin B12 levels with all-cause mortality in patients with MASLD, the main strengths of the current study are that it has a follow-up time of more than 20 years, adjusts for a variety of potential confounders, and takes into account the complex sampling design to enable the included samples to be more representative and to facilitate the generalization of the current findings. However, some limitations need to be considered. First, data on serum folate and vitamin B12 concentrations were based on a single measurement, so we were unable to assess the impact of dynamic changes in both concentrations on mortality. Second, because of the lack of histologic examination and natural history information, we were unable to analyze whether the effect of folate/vitamin B12 on mortality was confounded by the severity and duration of MASLD. Third, we did not exclude drug-induced hepatic steatosis because we could not establish a causal association between the two in the current cohort. Fourth, some of the non-statistically significant findings, especially in stratified analyses, may be related to the limited sample size. More studies are needed in the future to minimize type 2 error. Fifth, due to the limited availability of medication information, we cannot rule out the potential impact of drugs on vitamin B12 and folate levels. Additionally, the vitamin B12 profile of vegetarians may differ from that of the general population. Since we were unable to identify vegetarians within our cohort, it is unclear whether the current findings can be generalized to this group. Finally, despite our attempts to eliminate reverse causality by excluding participants who died within two years of follow-up, the current results are still not representative of a causal association between serum folate/vitamin B12 and mortality due to the inherent limitations of observational studies.

## Conclusions

Our results suggest a nonlinear association of serum folate and vitamin B12 levels with all-cause mortality in MASLD. Avoiding low serum folate and vitamin B12 concentrations may be potentially beneficial for reducing the risk of mortality in patients with MASLD.

## Data Availability

The original contributions presented in the study are included in the article/**Supplementary Material**. Further inquiries can be directed to the corresponding author.
