# Supplementary material for: Associations of serum folate and vitamin B12 levels with all-cause mortality among patients with metabolic dysfunction associated steatotic liver disease: a prospective cohort study
Source: Front Endocrinol (Lausanne). 2024 Dec 5;15:1426103. doi: 10.3389/fendo.2024.1426103 (PMC11655224; doi:10.3389/fendo.2024.1426103)
Supplement: Supplementary file 1 [file DataSheet1.docx]

**TABLE**

| **Supplementary Table 1.** Hazard ratios of All-Cause Mortality by Serum Folate and Vitamin B_12_ Levels Among Adults with MASLD (Excluding Participants Who Died Within Two Years of Follow-Up) | | | | | |
| --- | --- | --- | --- | --- | --- |
|  | **Age, sex, race-adjusted model ^a^** | |  | **Multivariable model ^b^** | |
|  | **HR (95% CIs)** | ***P-value*** |  | **HR (95% CIs)** | ***P-value*** |
| **Serum Folate** | | | | | |
| Quartile 1 | 1 (reference) |  |  | 1 (reference) |  |
| Quartile 2 | 0.75 (0.60-0.93) | 0.011 |  | 0.79 (0.60-1.04) | 0.098 |
| Quartile 3 | 0.68 (0.56-0.83) | <0.001 |  | 0.72 (0.56-0.93) | 0.011 |
| Quartile 4 | 0.73 (0.58-0.92) | 0.009 |  | 0.87 (0.67-1.14) | 0.314 |
| **Serum Vitamin B12** | | | | | |
| Quartile 1 | 1 (reference) |  |  | 1 (reference) |  |
| Quartile 2 | 0.81 (0.57-1.15) | 0.235 |  | 0.70 (0.50-0.99) | 0.046 |
| Quartile 3 | 0.77 (0.54-1.10) | 0.151 |  | 0.58 (0.39-0.88) | 0.009 |
| Quartile 4 | 0.91 (0.68-1.22) | 0.534 |  | 0.73 (0.55-0.97) | 0.029 |
| **Serum Folate & Vitamin B12*** | | | | | |
| Low folate & low vitamin B12 | 1 (reference) |  |  | 1 (reference) |  |
| Low folate & high vitamin B12 | 0.99 (0.68-1.46) | 0.974 |  | 0.83 (0.53-1.29) | 0.408 |
| High folate & low vitamin B12 | 0.79 (0.56-1.12) | 0.188 |  | 0.84 (0.59-1.21) | 0.344 |
| High folate & high vitamin B12 | 0.72 (0.53-0.99) | 0.045 |  | 0.64 (0.45-0.92) | 0.015 |
| ^a^ Multivariable Cox proportional regression analysis adjusted for age, sex, and race/ethnicity;  ^b^ Multivariable Cox proportional regression analysis adjusted for age, sex, race/ethnicity, educational level, marital status, family income level, smoking status, physical activity, Healthy Eating Index, FIB-4 index, serum triglycerides, C-reactive protein, body mass index, waist circumference, self-reported general health, diabetes mellitus, hypertension, history of heart attack, vitamin C intake, and vitamin E intake.  * Serum folate and serum vitamin B12 levels were considered simultaneously. Values below the median were classified as low levels, and values above the median were classified as high levels. Based on these two serum indicators, patients were divided into four groups: low folate & low vitamin B12 group, low folate & high vitamin B12 group, high folate & low vitamin B12 group, and high folate & high vitamin B12 group.  Abbreviation: MASLD, Metabolic dysfunction associated steatotic liver disease; HR, hazard ratio; CIs, confidence intervals | | | | | |

**FIGURE**

**
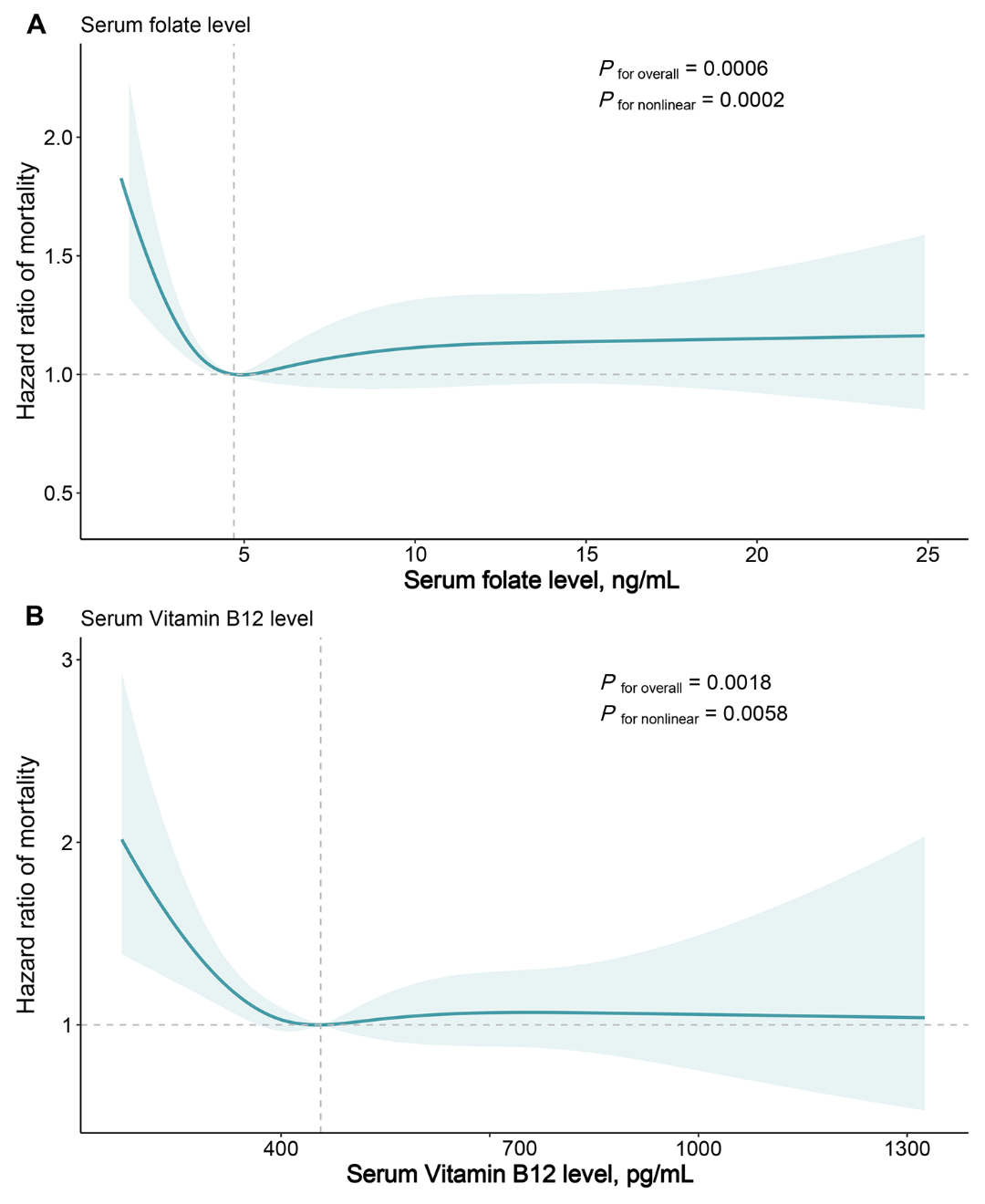
**

**Supplementary Figure 1.** Dose-response Association of Serum Folate (**A**) and Vitamin B_12_ (**B**) Levels with All-cause Mortality in Patients with Metabolic Dysfunction-associated Fatty Liver Disease (Excluding Participants Who Died Within Two Years of Follow-Up).

Hazard ratios were estimated by multivariable restricted cubic spline models, with knots placed at 5th, 35th, 65th, and 95th percentiles. Solid line represents hazard ratios and shaded areas represents 95% confidence intervals. The reference points are the median values for serum folate (4.7 ng/mL) and serum vitamin B12 (457.5 pg/mL) level. Risk estimates were adjusted for baseline age, sex, and race/ethnicity, educational level, marital status, family income level, smoking status, physical activity, Healthy Eating Index, FIB-4 index, serum triglycerides, C-reactive protein, body mass index, waist circumference, self-reported general health, diabetes mellitus, hypertension, and history of heart attack.
